# Molecular Mechanisms Underlying the Analgesic Property of Intrathecal Dexmedetomidine and Its Neurotoxicity Evaluation: An In Vivo and In Vitro Experimental Study

**DOI:** 10.1371/journal.pone.0055556

**Published:** 2013-02-07

**Authors:** Hongxing Zhang, Fang Zhou, Chen Li, Min Kong, He Liu, Peng Zhang, Song Zhang, Junli Cao, Licai Zhang, Hong Ma

**Affiliations:** 1 The First Clinical College, China Medical University, Shenyang, Liaoning, People’s Republic of China; 2 Department of Anesthesiology, The First Affiliated Hospital of China Medical University, Shenyang, Liaoning, People’s Republic of China; 3 Jiangsu Key Laboratory of Anesthesiology, Xuzhou Medical College, Xuzhou, Jiangsu, People’s Republic of China; 4 School of Nursing, Xuzhou Medical College, Xuzhou, Jiangsu, People’s Republic of China; 5 Department of Anesthesiology, The Affiliated Hospital of Xuzhou Medical College, Xuzhou, Jiangsu, People’s Republic of China; The Hebrew University Medical School, Israel

## Abstract

**Background:**

Dexmedetomidine (DEX) has been used under perioperative settings as an adjuvant to enhance the analgesic property of local anesthetics by some anesthesiologists. However, the analgesic mechanisms and neurotoxicity of DEX were poorly understood. This study examined the effect of DEX alone on inflammatory pain, and it also examined the underlying molecular mechanisms of DEX in the spinal cord. Furthermore, in vivo and in vitro experiments were performed to investigate the neurotoxicity of DEX on the spinal cord and cortical neurons.

**Methods:**

This study used adult, male Kunming mice. In the acute inflammatory model, the left hind-paws of mice were intradermally injected with pH 5.0 PBS while chronic constrictive injury (CCI) of the sciatic nerve was used to duplicate the neuropathic pain condition. Thermal paw withdrawal latency and mechanical paw withdrawal threshold were tested with a radiant heat test and the Von Frey method, respectively. Locomotor activity and motor coordination were evaluated using the inverted mesh test. Western blotting examined spinal ERK1/2, p-ERK1/2, caspase-3 and β-actin expressions, while spinal c-Fos protein expression was realized with immunohistochemical staining. Hematoxylin eosin (HE) staining was used to examine the pathological impacts of intrathecal DEX on the spinal cord. DAPI (4′,6-diamidino-2-phenylindole) staining was used to observe cell death under an immunofluorescence microscope.

**Results:**

Intra-plantar pH 5.0 PBS-induced acute pain required spinal ERK1/2 activation. Inhibition of spinal ERK1/2 signaling by intrathecal injection of DEX displayed a robust analgesia, via a α2-receptor dependent manner. The analgesic properties of DEX were validated in CCI mice. In vivo studies showed that intrathecal DEX has no significant pathological impacts on the spinal cord, and in vitro experiments indicated that DEX has potential protective effects of lidocaine-induced neural cell death.

**Conclusion:**

Intrathecal injection of DEX alone or as an adjuvant might be potential for pain relief.

## Introduction

For decades, clonidine, an α2-adrenergic receptor (α2-AR) agonist with a 220∶1 ratio of α2: α1 receptor binding, has been widely used as an analgesic adjuvant for pain therapy [1]. Nevertheless, a recent report demonstrated that α1-AR activity counterbalanced α2-AR-induced analgesia and, therefore, agonists with a higher α2-AR selectivity might be better choices for pain control [2]. In the 1990s, DEX, a newly developed α2-AR agonist with a 1620∶1 ratio of α2: α1 receptor binding (8∼10 fold stronger binding than clonidine), was first introduced into clinical practice as a short-term intravenous sedative in the intensive care unit [1]. Recently, studies have confirmed its potential as an adjuvant for pain treatment, mostly during the acute perioperative settings [1], which indicated DEX might act as a new drug in pain control. An early study by Eijs A. Kalso and colleagues has substantiated the antinociceptive property of DEX in rats, but the involved signal pathway still remains to be elucidated [3].

It is well established that extracellular signal-regulated protein kinase (ERK), a sub-family of the mitogen-activated protein kinases (MAPKs) family, contributes to different nociceptive processes and central sensitization induced by different noxious stimuli, such as capsaicin, formalin, carrageenan and Freund’s adjuvant [4]–[7]. Intrathecal inhibition of ERK1/2 phosphorylation attenuated nociceptive behaviors in different pain models. Spinal ERK signaling is activated through phosphorylation, and phosphorylated ERK has been considered as a marker of neuronal sensitization in a pain behavior-dependent manner [8]. These studies demonstrated an essential role of ERK1/2 in pain modulation. Moreover, ERK1/2 is densely expressed in the dorsal spinal cord [8]. Therefore, the present study was performed to examine the possible involvement of spinal ERK, a candidate signaling molecule, in a novel acute inflammatory pain model [9]. Our hypothesis was that activation of spinal α2-ARs by intrathecal DEX attenuates the intra-plantar acidic solution-induced pain behaviors via regulation of the spinal ERK signaling pathway. Some anesthesiologists have applied DEX intrathecally as an adjuvant to enhance the analgesic property of local anesthetics, however, the neurotoxicity of DEX on the nervous system was poorly known. To address this question, and in light of the higher chance of detecting toxicity as well as the potential use for chronic pain control, the pathological effects of repeated administration of intrathecal DEX into the spinal cord was tested in normal animals, as was the effect of DEX on local anesthetics-induced neural cell death, which was demonstrated by lidocaine administration.

## Animals and Methods

### Animals

The adult male Kunming mice, 18∼22 g, employed in the present study were provided by *Experimental Animal Center of China Medical University*. Mice were housed in standard transparent plastic cages, under a light-dark cycle (light at 08∶00 am), and with food and water *ad libitum*. Before experiments, the animals were allowed to habituate to the housing facilities for 7 days.

### Ethics Statement

All experimental protocols were approved by the Institutional Animal Care and Use Committees of China Medical University (IACUC-2010888) and enacted according to the guidelines of the International Association for the Study of Pain [10].

### Drugs

Phosphate Buffered Saline (PBS): NaCl 137 mM, KCl 2.7 mM, Na_2_HPO_4_ 10 mM, KH_2_PO_4_ 2 mM, was adjusted to pH 5.0 and 7.4 with dilute hydrochloric acid as described previously [9]. DEX and lidocaine were purchased from *Nhwa* Pharmaceutical Group(Jiangsu, China)and Sigma (St. Louis, Missouri), respectively. The MEK inhibitor (U0126) was obtained from Biomol Research Laboratories (Plymouth Meeting, PA) and dissolved in DMSO for storage. However, the final concentration of DMSO was less than 1%. Atipamezole, a specific antagonist of the α2 receptor, was provided by Suzhou *Yield Pharma* Co., Ltd (Jiangsu, China). All drugs were diluted with artificial cerebral spinal fluid (ACSF: NaCL 128 mM, D-Glucose 10 mM, NaH_2_PO_4_ 1.25 ml, NaHCO_3_ 24 mM, MgSO_4_ 2 mM, KCL 3 mM and CaCL_2_ 2 mM), resulting in a final volume of 10 µl. U0126 (0.5 µg) and atipamezole (1 µg) was applied intrathecally 30 min before the next injection. DEX pretreatment (0.04, 0.20 or 1.00 µg, if not mentioned is 1.00 µg) was given 5 min before the next injection. In vitro experiments used 1 mM lidocaine and 3 µM DEX.

### Intrathecal Injection

The intrathecal injection procedure followed the method of Hylden and Wilcoxon in 1980 [11]. Briefly, a stainless needle attached to a 25 µl micro-syringe was inserted between the L5 and L6 vertebrae of conscious mice. A sudden slight flick of the tail indicated the needle entered into the subarachnoid space. Ten microlitres of drug solution or vehicle was injected over a period of more than 30 s. After complete injection of drug, the needle was removed after a 15-second wait, to ensure retention. Twelve out of 414 mice were excluded because of motor dysfunction.

### Acute Inflammatory Pain Model

As we reported recently, mice were gently restrained and 10 µl of pH 5.0 PBS was injected into the hind-paw intradermally, using a 25-µl Hamilton syringe. The pain behaviors last for ∼20 min [9].

### Chronic Constrictive Injury Model (CCI model)

Mice were anesthetized with ketamine/xylazine cocktail (100 mg/kg/5 mg/kg, intraperitoneal injection). The left sciatic nerve was exposed at the mid-thigh level through a small incision, and constrictive injury was performed proximal to the trifurcation with three loose ligatures using a 5-0 silk thread (at a 1-mm interval). The incision was closed in layers, and the wound was treated with antibiotics (Neosporin).

### Thermal Hyperalgesia

The IITC Plantar Analgesia Meter (IITC Life Science Inc., CA) was used to measure paw withdrawal latency, as described previously [9], [12], [13]. Mice were placed in transparent acrylic enclosures (7 × 9 × 11 cm) on a glass floor, and allowed to acclimatize for 1 h in a temperature-controlled room (23±2°C). The radiant heat source was placed under the hindpaw, and the paw withdrawal latency (PWL) was recorded as the time from the start of the radiant heat stimulus to paw withdrawal or licking. The base line was set to 10∼12 s by adjusting the heat source intensity, and an automatic 20 s cutoff was used to prevent tissue damage.

### Mechanical Allodynia

Mechanical allodynia was assessed with electronic Von Frey filaments (IITC Life Science Inc., CA). Animals were placed in separate plastic box (20 ×25 ×15 cm) on a metal mesh floor and allowed to acclimate for 1 h. The filaments were presented perpendicularly to the plantar surface, and brisk withdrawal or paw flinching were considered as positive responses, the digital number presented on the monitor was recorded as the paw withdrawal threshold (PWT).

### Inverted Mesh Test

In accordance with recent reports [9], [14], mice were placed in the middle of a 20×25-cm inverted mesh and acclimatized to climb to the outside and over the edge of the mesh, and mice could climb on mesh with all four limbs before experiments. After injecting 10 µl of pH 7.4 PBS, pH 5.0 PBS or lidocaine(2%), mice were placed onto the mesh for a 1 min-observation (to prevent exhausted inability), and the time of four-paw holding was recorded.

### Western Blotting

Thirty micrograms of proteins were electrophoresed in a 10% sodium dodecyl sulfate (SDS)-polyacrylamide gel and transferred onto nitrocellulose membrane. The membranes were incubated at 4°C for 12 h with the primary polyclonal rabbit anti-p-ERK1/2, anti-ERK1/2, caspase-3 or β-actin antibody (1∶1,000, cell signaling technology, CST). The membranes were washed with Tris-Buffered Saline Tween-20 and incubated for 2 h with the secondary antibody conjugated with alkaline phosphatase (1∶500, Santa Cruz Technology, SCT) at room temperature. The immune complexes were detected using a nitro blue tetrazolium/5-bromo-4-chloro-3-indolyl phosphate assay kit (Sigma, St. Louis, MO). Western blot densitometry analysis was performed with Adobe Photoshop software (Adobe Systems Inc.), and gray-scale value of p-ERK1/2 were normalized to total ERK1/2, and caspase-3 to β-actin. The normalized density of control was set as 1. Spinal ERK1/2 and p-ERK1/2 were detected at the 10 min time point after pH 5.0 PBS treatment if not mentioned specially; caspase-3 and β-actin were detected at the 4 h time point after drug(s) incubation.

### Immunohistochemistry

Mice were anesthetized with ketamine/xylazine cocktail (100 mg/kg/5 mg/kg, intraperitoneal injection) and perfused intracardially with saline followed by 4% ice-cold paraformaldehyde in 0.1 M phosphate buffer. The L_4–5_ spinal cord were removed, post-fixed in 4% paraformaldehyde for 3 h at room temperature, and subsequently allowed to equilibrate in 30% sucrose in phosphate buffer overnight at 4°C. Thirty-µm transverse sections were cut on a cryostat. After washing in PBS, the tissue sections were blocked in PBS containing 5% normal goat serum and 0.3% TritonX-100 at room temperature for 1 h. For spinal c-Fos protein staining, the sections were incubated in primary polyclonal rabbit-anti-Fos antibody (1∶1000) (Santa Cruz Biotechnology, Santa Cruz, CA) overnight at 4°C, then followed by biotinylated goat anti-rabbit (1∶200) (Santa Cruz Biotechnology, Santa Cruz, CA) at 37°C for 2 h and in avidin-biotin-peroxidase complex (1∶100) (Vector Labs, Burlingame, CA) at 37°C for 2 h. Finally, the sections were treated with 0.05% diaminobenzidine for 5–10 min and rinsed with PBS to end the reaction, mounted on gelatin-coated slides, air-dried, dehydrated with 70%–100% alcohol, cleared with xylene, and cover-slipped for microscopic observation. The total number of positive neurons in spinal cord dorsal horn was counted from 5 sections in each animal. All positive neurons were counted without considering the intensity of the staining. The c-Fos protein was detected at the 2 h time point after pH 5.0 PBS treatment.

### Pathological HE Staining

Mice were perfused and the spinal cord was removed and post-fixed as described above in the immunohistochemistry methods. The spinal cord was then embedded in paraffin and prepared for coronal sections (5 µm thick) using a microtome. Sections were deparaffinized, ethanol-rehydrated, and then stained with HE staining assay, like a previous study described, for general morphological changes [15]. The HE staining was performed at the 6 h time point after every DEX injection.

### In vitro Experiments

The neural cells used in this study were cortical neurons harvested from 18-day-old embryonic SD rats. The neural cells were plated on 24 multi-well plates pre-coated with poly-L-lysine at a density of 10^5∼6^ cells/cm^2^ and fed with neurobasal media supplemented with B27 (1×) and glutamine (25 µM) in a 5% CO_2_ and 95% air incubator at 37°C. Cells were ready to received treatment of lidocaine, DEX or both for 4 h on day 10. Cell death was assessed by DAPI staining assay as a prior study described [16], and cell death rate [(dead cell number/total cell number)*100%] was calculated.

### Statistics

Graph PAD Prism version 5 software (Graph Pad Software Inc., San Diego, CA) was used to perform all the statistical analyses. Alteration of expression of the proteins detected and the behavioral responses to thermal and mechanical stimuli over time among groups were tested with one-way or two-way *ANOVA* with repeated measures followed by *Bonferroni post hoc* tests, respectively. All data are presented as the means
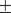
SEM. Statistical results are considered significant if *P*<0.05.

## Results

### Intra-plantar pH 5.0 PBS-induced Acute Pain Requires Spinal ERK1/2 Activation

**Figure 1:**
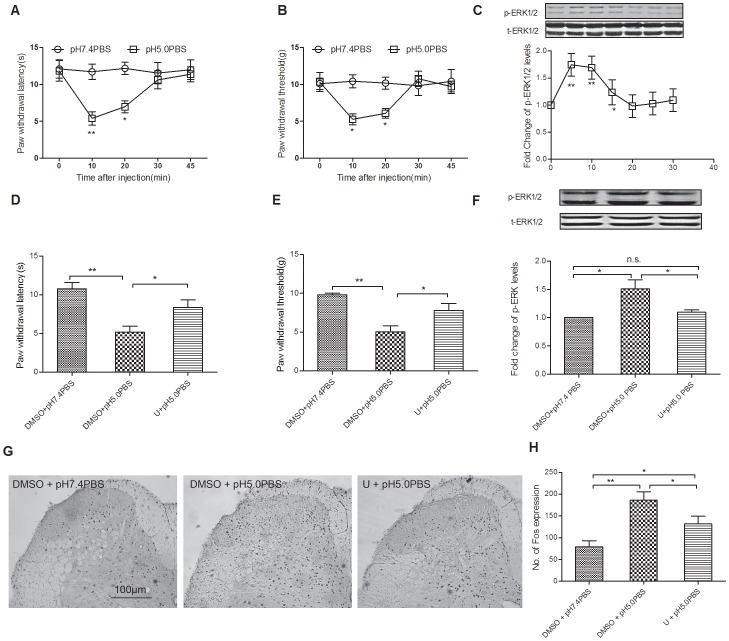
Acute inflammatory pain-induced by intradermal injection of pH 5.0 PBS requires spinal ERK1/2 phosphorylation. Intradermal injection of pH 5.0 PBS induced changes in thermal paw withdrawal latency, mechanical paw withdrawal threshold (A and B, **P<0.01 and *P<0.05, compared with pH 7.4 PBS group, n = 8), and spinal p-ERK1/2 expression(C, the representative bands and the quantitative data, **P<0.01 and *P<0.05, compared with 0 min time point). U0126 pretreatment inhibited the thermal withdrawal latency, mechanical withdraw threshold, and spinal p-ERK1/2 expression(D, E, F, **P<0.01, *P<0.05, n.s. means no significant difference, n = 8 for behavior tests and 6 for Western blotting tests) at 10 min time point, as well as spinal Fos protein at 2 h time point after pH 5.0 PBS injection.(G and F show the representative staining slices and the quantitative data, respectively, **P<0.01 and *P<0.05, n = 6, scale bar = 100 µm).

Intradermal injection of pH 5.0 PBS, but not pH 7.4 PBS, induced significant decrease of thermal paw withdrawal latency and mechanical paw withdrawal threshold for approximately 20 min (Fig. 1A and B). However, the underlying mechanisms remained unresolved [9]. Here we examined the expression of p-ERK1/2 in the lumbosacral enlargement at 0, 5, 10, 15, 20, 25 and 30 min after intradermal injection, and Western blotting analysis revealed a significant activation of p-ERK1/2 in parallel with the time course of behavioral results (Fig. 1D). Intrathecal pretreatment with U0126 (an inhibitor of MEK) reversed the pain behaviors (Fig. 1D and E), the increase of spinal p-ERK1/2 (Fig. 1F) and the increase in spinal c-Fos protein, a marker for neuronal activation (Fig. 1G and H).

### Intrathecal DEX Displays Analgesic Effect in a α2-receptor Dependent Manner via Spinal p-ERK1/2 Inhibition

**Figure 2:**
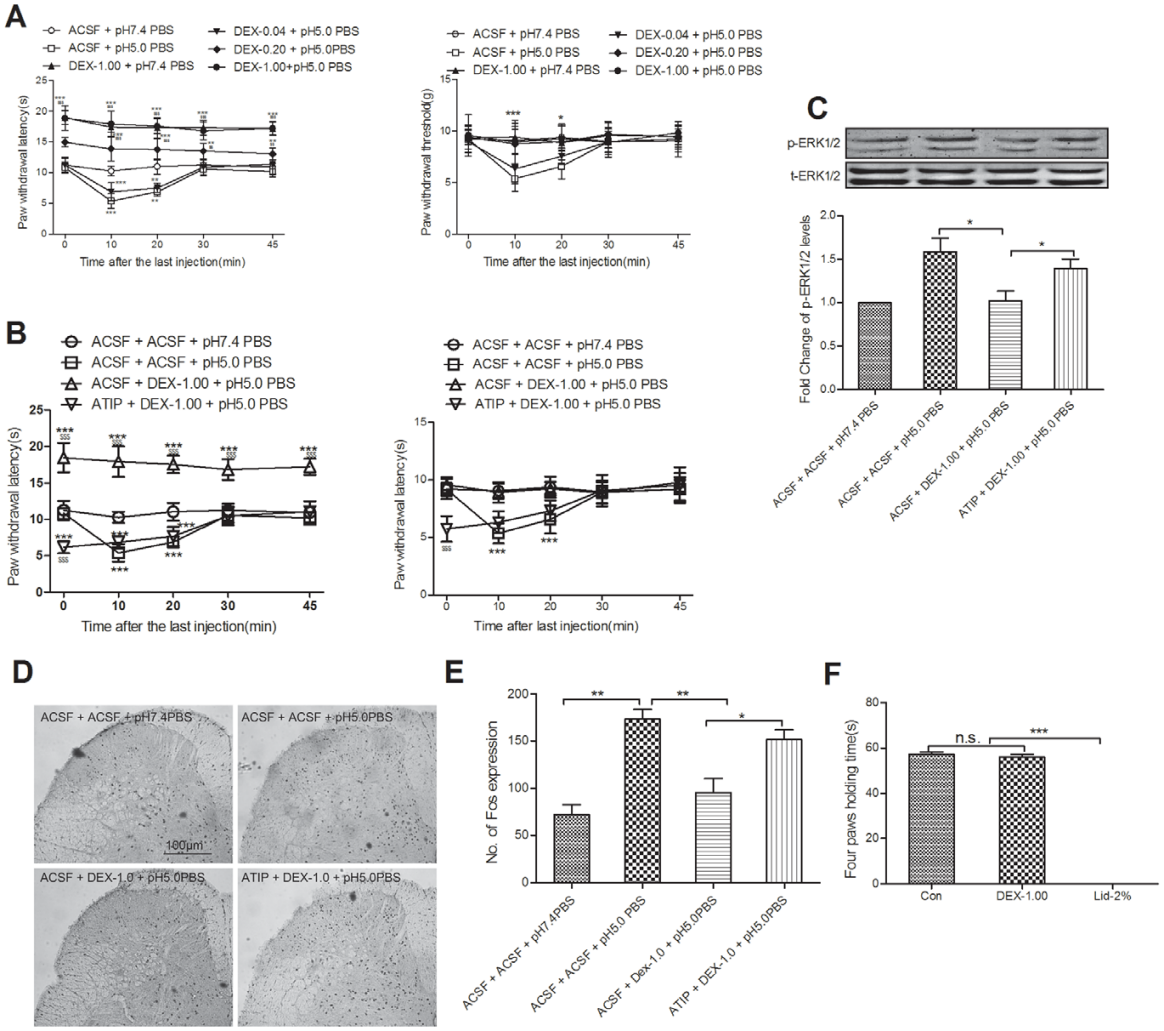
Intrathecal DEX shows analgesic property in a α2-R dependent manner via inhibiting spinal ERK1/2 phosphorylation. (A) Pretreatment with different doses of DEX increased the thermal withdrawal latency and mechanical withdrawal threshold in pH 5.0 PBS mice (***P<0.001, **P<0.01 compared with ACSF+pH 7.4 PBS group, $$$*P<*0.001, $$*P*<0.01, compared with ACSF+pH 5.0 PBS group, n = 8). Pretreatment with atipamezole blocks the effects of intrathecal DEX on pain behaviors (B: ***P<0.001, compared with ACSF+pH 7.4 PBS group, $$$*P<*0.001, compared with ACSF+PH5.0PBS group, n = 8), spinal p-ERK1/2(C: *P<0.05, n = 6) and c-Fos protein expression (D: **P<0.01, *P<0.05, scale bar = 100 µm, n = 6). (F) Four paws-holding time 1 minute after intrathecal DEX for 1 min, ***P<0.001, n.s. for no significance, n = 10.

To clarify the analgesic property of DEX, three doses of DEX (0.04, 0.20 and 1.00 µg) were investigated (for time points, see methods), and we found that DEX significantly increased the thermal paw withdrawal latency, even in control mice. The mechanical paw withdrawal threshold was increased in pain model mice but not in control mice (Fig. 2A). A previous study reported that intrathecal DEX showed analgesic effects in rats in an α2-ARs dependent manner while the downstream mechanisms remained unclear [3]. In the present study, we found that blocking spinal α2-ARs by intrathecal pretreatment with atipamezole (a selective α2-AR antagonist) reversed the DEX pretreatment-induced down-regulation of the thermal paw withdrawal latency and mechanical paw withdrawal threshold(Fig. 2B), and the up-regulation of spinal p-ERK1/2 (Fig. 2C) and the spinal c-Fos protein expression in pH 5.0 PBS mice (Fig. 1D and E).

The motor function was evaluated with the inverted mesh test, as previously described [9]. The results showed that intrathecal injection of DEX had no effect on the four-paw holding time compared with control group, while mice that received lidocaine (2%, 10 µl) could not hold the mesh with their hind-paws (Fig. 2F). These data indicated that spinal ERK1/2 signaling inhibition contributed to the analgesia of intrathecal DEX induced α2-ARs activation without impairing the motor function.

### Intrathecal DEX Inhibits Chronic Neuropathic Pain

**Figure 3:**
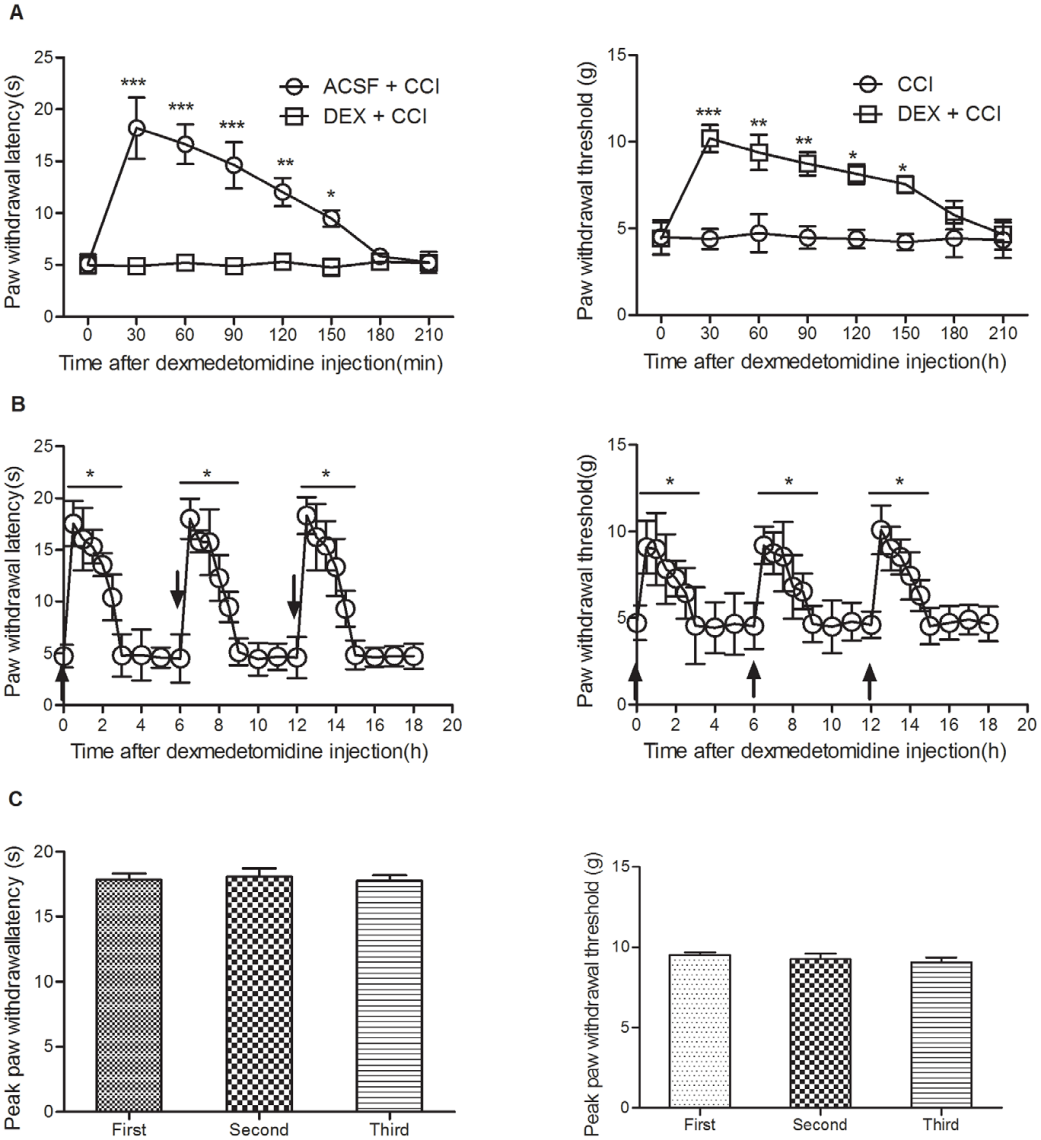
Intrathecal DEX inhibits CCI-induced persistent pain. Intrathecal DEX increased the thermal paw withdrawal latency and mechanical paw withdrawal threshold (A, ***P<0.001,**P<0.01 and *P<0.05, compared with 0 min time point, n = 8). Repeated intrathecal *DEX* displays similar analgesic property both in analgesic duration (B, *P<0.05, compared with 0 min time point, n = 8, arrows indicate *i.t*. injection time point) and intensity(C).

On day 7 of CCI, mice received three intrathecal injections of DEX, one injection every 6-hour interval. After each injection, thermal and mechanical pain behaviors were observed for 6 hours. The results demonstrated that the analgesic property of each injection had a similar time-course and intensity (Fig. 3 A, B and C). These data suggested that intrathecal DEX also had an analgesic property in treating chronic neuropathic pain without acute tolerance.

### Pathological Effects of Intrathecal DEX on Spinal Cord

**Figure 4:**
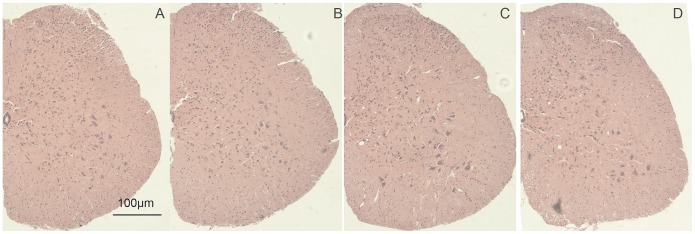
Pathological effect of repeated administration of DEX intrathecally on spinal cord in control mice. A∼D show HE-staining results 6 h after mice received 0, 1, 2, 3 times of intrathecal DEX (at a 6 h-interval, n = 4, scale bar = 100 µm).

To address pathological effects, DEX was injected intrathecally once, twice or three times (injection/6 h) in control mice, and HE staining was performed to observe DEX’s pathological property on the spinal cord at the 6 h time point after the last injection. No significant pathological injuries were observed when compared with control group (Fig. 4).

### Protective Effects of DEX on Lidocaine-induced Cell Death in Rat Cortical Neurons

**Figure 5:**
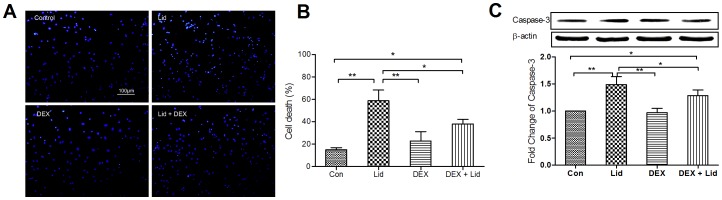
Protective effect of DEX on lidocaine-induced cortical neuron death in rat. Co-incubation of DEX reduced the cell death (A and B shown the representative DAPI staining results and the quantitative data, **P<0.01, *P<0.05, n = 10 visual fields, scale bar = 100 µm) and caspase-3 expression (C, **P<0.01, *P<0.05) induced by lidocaine after a 4 h-incubation.

When co-incubated with a cocktail of DEX and lidocaine, the cell death rate decreased significantly when compared with the lidocaine alone group, but was still higher than the cell death rate in control group(Fig. 5 A and B). DEX also reduced the lidocaine-induced caspase-3 expression, a caspase responsible for proteolytic cleavage that leads to cell disassembly[17]–[19]. While reduced, caspase-3 expression was still higher than the control (Fig. 5 C). No significance in β-actin expression was observed in different groups.

## Discussion

Studies reported that low pH solutions induced nociception both in animal and human beings [20], [21]. We found intradermal injection of acidic pH 5.0 PBS in the hind-paw induced pain behaviors immediately, peaked at 10 min. and subsided to baseline at approximately 30 min. Contrarily, Ries et al. reported acidic solutions were easily buffered shortly after administration into tissues [22]. We believe that 10 µl of acidic solution requires several minutes to complete buffering, allowing activated downstream mechanisms to contribute to the process of the following thermal and mechanical pain behaviors. Differences in research methods or variation of species might also account for the conflicting results.

Spinal ERK1/2 was a key signal molecule involved in different nociceptive processing (see introduction), as well as cell survival, synapse plasticity and the like [13], [23]. Therefore spinal ERK1/2 was chosen as the candidate in this study. As expected and in tune with the behavioral tests, results showed that intradermal injection of pH 5.0 PBS induced spinal ERK1/2 phosphorylation, which could be inhibited by intrathecal U0126 pretreatment. These data indicated that the spinal ERK1/2 signal pathway was involved in the acute inflammatory nociception induced by acidic solution. Next, the analgesic property of intrathecal DEX was investigated. Three different doses of DEX were administered 5 min before pH 5.0 PBS was given, and a long-lasting dose-dependent analgesic property was observed. As we thought, pretreatment with DEX also significantly reduced the activation of spinal ERK1/2 from acidic solution application. Behavioral and molecular changes induced by intrathecal DEX were prevented by atipamezole pretreatment, a selective α2-AR antagonist. This substantiated that intrathecal DEX induced spinal α2-AR activation, which displayed analgesic properties via inhibiting ERK1/2 signaling. Interestingly, U0126 or DEX only returned the paw withdrawal latency and threshold to the base line, while DEX, but not U0126, reversed the thermal paw withdrawal latency to a much higher level than the control. Therefore we believed that the analgesic property of intrathecal DEX was associated with pain styles. However, the effects of α2-ARs on ERK1/2 signaling can vary. For example, in other pathological processes, activation of α2-AR increased p-ERK1/2 expression, and sometimes, DEX did not work in a α2AR-dependent manner [24]. Differences in location and pathological processes might account for these variations and similar phenomena have been observed in many other pathological processes, and the effects of CREB and BDNF in mood regulation have displayed different, even inverted expressions in different brain regions such as the hippocampus and the mesolimbic system. In addition, antidepressants antagonized their lower or higher expressions in these different brain regions [25]. Due to the sedative effects of DEX, it was not surprising to observe obvious sedation in DEX-treated mice. A recent study thought the sedative property was due to intrathecally injected DEX circulating with the cerebral spinal fluid, and being transported to some α2-ARs enriched brain regions. In addition, DEX had general effects after being absorbed into the blood circulation [1].

For more than 30 years, spinal Fos protein has been considered as the marker of neuronal sensitization [26]; therefore, the expression of spinal c-Fos was tested to confirm the effective of treatments applied in this study.

To exclude the effects of intrathecal DEX on motive neurons in the spinal ventral horn, motor ability was assessed, but no positive results were observed. The results show that mice under sedation can still participate in reflex testing induced by noxious stimuli. Clinical study indicated that though the elimination half-life of DEX is short (2∼3 hours), it has a long-lasting analgesic-sparing effect (∼24 hours). Clinicians thought the anxiolytic, sedative, and thymoanaleptic properties of DEX might be, at least partly, responsible for this long-lasting analgesic effect [1]. These reasons cannot be excluded in this study. A powerful analgesic property was also found in persistent neuropathic pain mice, and no declining of analgesic time-course and intensity were observed when administered repeatedly. Taken together, the data indicated that intrathecal DEX had a large potential to control pain conditions in different categories. It was well known that drugs administered adjacent to the spinal cord or nerves might have the possibility to induce neurotoxicity especially when applied repeatedly or at a high concentration. In the present study, we found no significant pathological injuries after intrathecal DEX-treatment in control mice, even after repeated exposure. Nevertheless, one paper reported that DEX might have a harmful effect on the myelin sheath when administered via the epidural route and higher dose and concentration might account for the harm. Therefore, lower dose and concentration should be taken when used via epidural route [27]. We believe that the following reasons might partly account for the difference of these results: first, different administered routes were used (intrathecal injection and epidural catheter). Normally, a much larger dose and volume of drugs is given via an epidural route than given via an intrathecal manner, for example 5∼10 folds [27]. Second, the epidural space makes it difficult for drug solutions to diffuse. Therefore, almost all the drugs were absorbed and metabolized within the limited space, while the CSF actually plays a role in diluting the drugs applied into the subarachnoid space via the intrathecal method [1]. Species differences may also play a role in the observed discrepancies (mice and rabbits).

DEX was used as adjuvant to enhance the analgesic property of local anesthetics, such as lidocaine, bupivacaine and ropivacaine in perioperative conditions. However, in vivo and in vitro studies indicated that these local anesthetics had significant neurotoxicity [17], [28]. DEX showed protective or growth-promoting properties in many tissues, including nerve cells from the cortex [29], [30]. This evidence indicated that DEX has a potential protective property on local anesthetic-induced neurotoxicity. Taking lidocaine as an example, we found co-incubation of DEX decreased lidocaine-induced cortical neuron death significantly, though not to a normal level. Consistently, the expressions of caspase-3, a product in lidocaine and other local anesthetic-induced neurotoxicity that have been used as a mark of cell death [18], was reduced also. These data suggested that DEX might be helpful in reducing the neurotoxicity induced by local anesthetics.

In conclusion, this study highlights that activation of the ERK1/2 signaling pathway in the dorsal spinal cord contributes to the nociceptive behaviors induced by intraplantar pH 5.0 PBS, and in which activation of spinal α2-AR by intrathecal injection of DEX displayed strong analgesic property via inhibition of spinal ERK1/2 signaling pathway. And of particular to note is that no significant pathological effects of DEX on spinal cord was observed at cellular level, and in vitro experiments indicated that DEX might act as a preventer of the local anesthetics-induced neurotoxicity when used together with local anesthetics. Taken together, intrathecal dexmedetomidine might be potential to be used for pain relief.

## References

[1] Grosu I, Lavand'homme P (2010) Use of dexmedetomidine for pain control. F1000 Med Rep 2: 90. 21283652 10.3410/M2-90 PMC3026617

[2] Brummett CM, Trivedi KA, Dubovoy AV, Berland DW (2009) Dexmedetomidine as a novel therapeutic for postoperative pain in a patient treated with buprenorphine. J Opioid Manag 5: 175–179. 19662927 10.5055/jom.2009.0018

[3] Kalso EA, Poyhia R, Rosenberg PH (1991) Spinal antinociception by dexmedetomidine, a highly selective alpha 2-adrenergic agonist. Pharmacol Toxicol 68: 140–143. 1677190 10.1111/j.1600-0773.1991.tb02052.x

[4] Hwang YP, Yun HJ, Choi JH, Han EH, Kim HG, et al (2011) Suppression of EGF-induced tumor cell migration and matrix metalloproteinase-9 expression by capsaicin via the inhibition of EGFR-mediated FAK/Akt, PKC/Raf/ERK, p38 MAPK, and AP-1 signaling. Mol Nutr Food Res 55: 594–605. 21462327 10.1002/mnfr.201000292

[5] Lee MJ, Shin TJ, Lee JE, Choo H, Koh HY, et al (2010) KST5468, a new T-type calcium channel antagonist, has an antinociceptive effect on inflammatory and neuropathic pain models. Pharmacol Biochem Behav 97: 198–204. 20678515 10.1016/j.pbb.2010.07.018

[6] Sawatzky DA, Willoughby DA, Colville-Nash PR, Rossi AG (2006) The involvement of the apoptosis-modulating proteins ERK 1/2, Bcl-xL and Bax in the resolution of acute inflammation in vivo. Am J Pathol 168: 33–41. 16400007 10.2353/ajpath.2006.050058 PMC1592663

[7] Tashiro A, Okamoto K, Bereiter DA (2009) Chronic inflammation and estradiol interact through MAPK activation to affect TMJ nociceptive processing by trigeminal caudalis neurons. Neuroscience 164: 1813–1820. 19786077 10.1016/j.neuroscience.2009.09.058 PMC2813765

[8] Gao YJ, Ji RR (2009) c-Fos and pERK, which is a better marker for neuronal activation and central sensitization after noxious stimulation and tissue injury? Open Pain J 2: 11–17. 19898681 10.2174/1876386300902010011 PMC2773551

[9] Liu H, Zhang HX, Hou HY, Lu XF, Wei JQ, et al. (2011) Acid solution is a suitable medium for introducing QX-314 into nociceptors through TRPV1 channels to produce sensory-specific analgesic effects. PLoS One. United States: 2011 Liu, et al. pp. e29395. 10.1371/journal.pone.0029395 PMC3247264 22216270

[10] Zimmermann M (1983) Ethical guidelines for investigations of experimental pain in conscious animals. Pain 16: 109–110. 6877845 10.1016/0304-3959(83)90201-4

[11] Hylden JL, Wilcox GL (1980) Intrathecal morphine in mice: a new technique. Eur J Pharmacol 67: 313–316. 6893963 10.1016/0014-2999(80)90515-4

[12] Guan XH, Lu XF, Zhang HX, Wu JR, Yuan Y, et al. (2010) Phosphatidylinositol 3-kinase mediates pain behaviors induced by activation of peripheral ephrinBs/EphBs signaling in mice. Pharmacol Biochem Behav. United States: 2010 Elsevier Inc. 315–324. 10.1016/j.pbb.2010.02.007 20170671

[13] Ruan JP, Zhang HX, Lu XF, Liu YP, Cao JL (2010) EphrinBs/EphBs signaling is involved in modulation of spinal nociceptive processing through a mitogen-activated protein kinases-dependent mechanism. Anesthesiology 112: 1234–1249. 20395829 10.1097/ALN.0b013e3181d3e0df

[14] Leszczynska K, Kau ST (1992) A sciatic nerve blockade method to differentiate drug-induced local anesthesia from neuromuscular blockade in mice. J Pharmacol Toxicol Methods 27: 85–93. 1591408 10.1016/1056-8719(92)90026-w

[15] Li LY, Li JL, Zhang HM, Yang WM, Wang K, et al. (2012) TGFbeta1 Treatment Reduces Hippocampal Damage, Spontaneous Recurrent Seizures, and Learning Memory Deficits in Pilocarpine-Treated Rats. J Mol Neurosci. 10.1007/s12031-012-9879-1 22936246

[16] Lee YJ, Park HH, Koh SH, Choi NY, Lee KY (2011) Amlodipine besylate and amlodipine camsylate prevent cortical neuronal cell death induced by oxidative stress. J Neurochem 119: 1262–1270. 21988238 10.1111/j.1471-4159.2011.07529.x

[17] Werdehausen R, Fazeli S, Braun S, Hermanns H, Essmann F, et al (2009) Apoptosis induction by different local anaesthetics in a neuroblastoma cell line. British Journal of Anaesthesia 103: 711–718. 19700777 10.1093/bja/aep236

[18] Werdehausen R, Braun S, Essmann F, Schulze-Osthoff K, Walczak H, et al (2007) Lidocaine induces apoptosis via the mitochondrial pathway independently of death receptor signaling. Anesthesiology 107: 136–143. 17585225 10.1097/01.anes.0000268389.39436.66

[19] Perez-Castro R, Patel S, Garavito-Aguilar ZV, Rosenberg A, Recio-Pinto E, et al. (2009) Cytotoxicity of local anesthetics in human neuronal cells. Anesth Analg. United States. 997–1007. 10.1213/ane.0b013e31819385e1 19224816

[20] Drizin I, Gomtsyan A, Bayburt EK, Schmidt RG, Zheng GZ, et al (2006) Structure-activity studies of a novel series of 5,6-fused heteroaromatic ureas as TRPV1 antagonists. Bioorg Med Chem 14: 4740–4749. 16621571 10.1016/j.bmc.2006.03.027

[21] Jones NG, Slater R, Cadiou H, McNaughton P, McMahon SB (2004) Acid-induced pain and its modulation in humans. J Neurosci 24: 10974–10979. 15574747 10.1523/JNEUROSCI.2619-04.2004 PMC6730214

[22] Ries CR, Pillai R, Chung CC, Wang JT, MacLeod BA, et al (2009) QX-314 produces long-lasting local anesthesia modulated by transient receptor potential vanilloid receptors in mice. Anesthesiology 111: 122–126. 19512885 10.1097/ALN.0b013e3181a9160e

[23] Mendoza MC, Er EE, Blenis J (2011) The Ras-ERK and PI3K-mTOR pathways: cross-talk and compensation. Trends Biochem Sci. England: 2011 Elsevier Ltd. 320–328. 10.1016/j.tibs.2011.03.006 PMC3112285 21531565

[24] Dahmani S, Paris A, Jannier V, Hein L, Rouelle D, et al (2008) Dexmedetomidine increases hippocampal phosphorylated extracellular signal-regulated protein kinase 1 and 2 content by an alpha 2-adrenoceptor-independent mechanism: evidence for the involvement of imidazoline I1 receptors. Anesthesiology 108: 457–466. 18292683 10.1097/ALN.0b013e318164ca81

[25] Nestler EJ, Carlezon WA Jr (2006) The mesolimbic dopamine reward circuit in depression. Biol Psychiatry 59: 1151–1159. 16566899 10.1016/j.biopsych.2005.09.018

[26] Hunt SP, Pini A, Evan G (1987) Induction of c-fos-like protein in spinal cord neurons following sensory stimulation. Nature 328: 632–634. 3112583 10.1038/328632a0

[27] Konakci S, Adanir T, Yilmaz G, Rezanko T (2008) The efficacy and neurotoxicity of dexmedetomidine administered via the epidural route. Eur J Anaesthesiol 25: 403–409. 18088445 10.1017/S0265021507003079

[28] Sites BD, Taenzer AH, Herrick MD, Gilloon C, Antonakakis J, et al (2012) Incidence of local anesthetic systemic toxicity and postoperative neurologic symptoms associated with 12,668 ultrasound-guided nerve blocks: an analysis from a prospective clinical registry. Reg Anesth Pain Med 37: 478–482. 22705953 10.1097/AAP.0b013e31825cb3d6

[29] Bruzzone A, Pinero CP, Castillo LF, Sarappa MG, Rojas P, et al (2008) Alpha2-adrenoceptor action on cell proliferation and mammary tumour growth in mice. Br J Pharmacol 155: 494–504. 18604234 10.1038/bjp.2008.278 PMC2579667

[30] Sanders RD, Sun P, Patel S, Li M, Maze M, et al (2010) Dexmedetomidine provides cortical neuroprotection: impact on anaesthetic-induced neuroapoptosis in the rat developing brain. Acta Anaesthesiol Scand 54: 710–716. 20003127 10.1111/j.1399-6576.2009.02177.x

